# Comparative Untargeted Metabolomics of Flavonoid- and Phenylpropanoid-Related Differential Metabolites in Different Tissues and Growth Stages of *Apocynum venetum* and *Apocynum pictum*

**DOI:** 10.3390/molecules31193401

**Published:** 2026-09-24

**Authors:** Wei-Jian Huang, Yu-Qing Wang, Hua-Liang Zhang, Jian-Min Cong

**Affiliations:** 1 School of Smart Agricultural Engineering, Taizhou Vocational College of Science & Technology, Taizhou 318020, China; huangweijian@tzkjzyxy.wecom.work (W.-J.H.); 19512322618@163.com (Y.-Q.W.); hualiangzhang@zju.edu.cn (H.-L.Z.); 2 Taizhou Key Laboratory of Horticultural Biotechnology, Taizhou 318020, China; 3 Institute of Soil and Water Resources and Environmental Science, Zhejiang University, Hangzhou 310058, China

**Keywords:** *Apocynum venetum*, *Apocynum pictum*, metabolomics, flavonoid, phenylpropanoid metabolites, LC-MS/MS

## Abstract

*Apocynum venetum* and *Apocynum pictum* are two important medicinal plants of the same genus but distinct species; their flavonoid and phenylpropanoid constituents are closely related to their medicinal value. Untargeted metabolomics was used to compare the flavonoid- and phenylpropanoid-related differential metabolites of roots, stems, and leaves in the two species at the seedling, growth, and mature stages. In total, 568, 560, and 530 differential metabolites were annotated in roots, stems, and leaves, respectively, of which 39 were related to the flavonoid and phenylpropanoid pathways. KEGG enrichment analysis showed that flavonoid and phenylpropanoid biosynthesis were enriched in leaves and flavone and flavonol biosynthesis in stems. Among the annotated metabolites, flavone/flavonol-type compounds (Quercetin and Luteolin) and phenylpropanoid compounds (Sinapic acid, Sinapyl alcohol, Caffeic acid, 5-caffeoylshikimic acid) differed markedly between species and among tissues, whereas flavanones and their derivatives, the flavanonol Taxifolin (Level 2) and naringenin-O-dihexoside (Level 3), were enriched in *A. pictum*. Overall, species, tissue, and growth stage jointly influence the accumulation of flavonoid- and phenylpropanoid-related metabolites in *Apocynum*, providing a basis for germplasm evaluation, medicinal-material identification, and the rational utilization of different tissues.

## 1. Introduction

The genus *Apocynum* L. (Apocynaceae) comprises perennial herbaceous plants. *Apocynum venetum* L. and *Apocynum pictum* are two closely related species of the same genus. They are important wild medicinal plant resources in China, widely distributed and genetically diverse [1,2]; however, the differences in their medicinal components remain incompletely understood, and a systematic comparison of their chemical profiles is an essential basis for species identification and quality evaluation. The leaves of *A. venetum* are included in the Chinese Pharmacopoeia; they are commonly processed into health beverages such as *Apocynum* tea [3,4]. Flavonoids are among the main bioactive components [5], exhibiting antihypertensive, sedative, antidepressant, antioxidant, and anti-inflammatory activities [6,7,8]. In addition to the leaves designated for medicinal use by the Pharmacopoeia, the roots and stems also accumulate bioactive components such as flavonoids, and differences in their accumulation among species, tissues, and growth stages are directly related to the material basis of their pharmacological activity and the value of whole-plant utilization. The abundance and composition of flavonoids therefore directly determine the medicinal and health-promoting value of *Apocynum*, making them a central target for chemical evaluation of this genus.

In addition to flavonoids, the phenylpropanoid pathway is a major source of bioactive plant natural products. It gives rise to a wide range of compounds—phenolic acids, coumarins, monolignols and their glucosides, and lignans—many of which exhibit antioxidant, anti-inflammatory, antimicrobial, hepatoprotective, or phytoestrogenic activities [9,10,11]. In Apocynum, phenylpropanoid and monolignol derivatives, such as chlorogenic acid, Caffeic acid, Sinapic acid, and the monolignol glucoside Coniferin, co-accumulate with flavonoids and contribute to the pharmacological and sensory properties of Apocynum tea and related preparations [8,12]. Nevertheless, phenylpropanoid-related metabolites have rarely been compared systematically between *A. venetum* and *A. pictum* across tissues and developmental stages, and their contribution to the interspecific metabolomic differences remains largely unexplored.

Current studies are limited to individual tissues [13,14,15] or focus only on the total flavonoid content at harvest [16,17]; comparative studies of flavonoid- and phenylpropanoid-related differential metabolites across roots, stems, and leaves and across multiple growth stages in *A. venetum* and *A. pictum* are rarely reported, and species identification still lacks distinctive chemical markers, which hampers the differentiated utilization of *Apocynum* roots and stems. Metabolomics can comprehensively characterize the chemical phenotype of plants at the metabolite level and has been widely applied to species identification and quality evaluation of medicinal plants.

Therefore, in this study, LC-MS/MS-based untargeted metabolomics was used to compare flavonoid- and phenylpropanoid-related differential metabolites in the roots, stems and leaves of the two species at the seedling, growth and mature stages. We analyzed their pathway-enrichment characteristics and tissue- and stage-dependent distribution and screened candidate characteristic metabolites with stable between-species differences. This provided a metabolomic basis for the quality evaluation, species identification and differentiated utilization of whole-plant *Apocynum* resources. In this study, metabolite annotation was performed by matching MS and MS/MS spectra against the HMDB v5.0, Metlin v3.0, and the in-house plant-specific metabolite database (MJDBPM) of Majorbio, with a mass error below 10 ppm and MS/MS spectral matching scores. 

## 2. Results

### 2.1. Quality Assessment of the Metabolomics Data

The analytical run passed quality control. Pooled QC samples were analyzed alongside the samples. In the cumulative RSD distribution of the QC peaks (Figure 1a), 81.09% of the raw features had a relative standard deviation (RSD) below 30%, and data preprocessing further reduced the RSD (Figure 1a). The base-peak chromatograms (BPC) in both positive and negative ESI modes showed consistent peak shapes and retention times across the QC and sample runs (Figure 1b), confirming chromatographic and instrumental stability. Samples clustered primarily by tissue (PC1 = 32.90%, PC2 = 14.90%; Figure 1c), and within each tissue, separation between *A. venetum* and *A. pictum* was revealed by supervised PLS-DA (R^2^Y = 0.671, Q^2^ = 0.522, 7-fold cross-validation; Figure 1d) and ANOSIM (R = 0.7237, *p* = 0.001).

A total of 5038 metabolites were detected by LC-MS/MS, including 3289 primary metabolites and 1749 secondary metabolites. Among all metabolites, 2378, 2536, and 2918 were detected in roots, stems, and leaves, respectively. The major classes of primary and secondary metabolites are shown in Table 1.

### 2.2. Screening of Differential Metabolites in Roots, Stems, and Leaves

*A. venetum* was set as the reference species: log_2_FC > 0 indicates higher abundance in *A. venetum* (upregulated), and log_2_FC < 0 indicates higher abundance in *A. pictum* (downregulated). Using VIP > 1, *p* < 0.05, and |log_2_FC| ≥ 1 as the screening criteria, 568 differential metabolites (174 up-regulated and 394 down-regulated) were annotated in roots, 560 (306 up-regulated and 254 down-regulated) in stems, and 530 (284 up-regulated and 246 down-regulated) in leaves. Differential metabolites in roots were predominantly down-regulated (69.4%), whereas slightly more up-regulated than down-regulated metabolites were observed in stems and leaves (Figure 2a–d). The prevalence of down-regulated metabolites in roots indicates that a large proportion of root differential metabolites were more abundant in *A. pictum*, whereas the slightly higher numbers of up-regulated metabolites in stems and leaves indicate that the two species contributed comparably to the metabolome of these aerial tissues.

In roots, the upregulated metabolites belonged mainly to O-glycosyl compounds (10), fatty acyls (9), cinnamic acid and derivatives (8), benzopyrans (6), and flavonoid glycosides (5). The down-regulated metabolites were mainly fatty acyls (28), coumarins and derivatives (18), benzoic acid and derivatives (17), flavonoid glycosides (16) and alpha amino acids and derivatives (15).

In stems, the up-regulated metabolites mainly belonged to flavonoid glycosides (24), fatty acyls (20), O-glycosyl compounds (16), coumarins and derivatives (10), and benzoic acid and derivatives (8). The down-regulated metabolites were mainly fatty acyls (31), alpha amino acids and derivatives (14), organic acids and derivatives (10), ketones and derivatives (9) and benzoic acid and derivatives (6).

In leaves, the up-regulated metabolites belonged mainly to flavonoid glycosides (29), coumarins and derivatives (13), fatty acyls (11), cinnamic acid and derivatives (9), and benzopyrans (8). The down-regulated metabolites were mainly fatty acyls (30), alpha amino acids and derivatives (13), organic acids and derivatives (11), O-glycosyl compounds (9) and benzoic acid and derivatives (9). Overall, these compositional differences were dominated by phenylpropanoid- and flavonoid-related classes, consistent with the central role of flavonoid metabolism in shaping the interspecific metabolomic divergence.

### 2.3. KEGG Pathway Enrichment Analysis of Differential Metabolites

KEGG pathway enrichment analysis was performed to clarify the biological functions of the differential metabolites. The results showed that the main enriched pathways differed markedly among tissues.

The enriched pathways in roots included alanine, aspartate, and glutamate metabolism; phenylalanine, tyrosine, and tryptophan biosynthesis; flavonoid biosynthesis; biosynthesis of various plant secondary metabolites; and galactose metabolism, with the largest number of differential metabolites enriched in the ‘biosynthesis of various plant secondary metabolites’ pathway (Figure 3a).

The most significantly enriched pathways in stems included aminoacyl-tRNA biosynthesis, ABC transporters, D-amino acid metabolism, and flavone and flavonol biosynthesis, with 13 metabolites enriched in the ABC transporter pathway (Figure 3b).

The most significantly enriched pathways in leaves included flavonoid biosynthesis, the biosynthesis of various plant secondary metabolites, and phenylpropanoid biosynthesis (Figure 3c).

### 2.4. VIP Analysis of Differential Metabolites

The VIP value of each differential metabolite was calculated to identify the key metabolites contributing to the distinction between the two species. The metabolites with the highest VIP values differed among tissues, and flavonoid compounds such as Tiliroside and Apigenin 7-O-(6″-malonyl-apiosyl-glucoside) showed high VIP values in the corresponding tissues (Figure 4a–c). The high VIP values of these flavonoid compounds indicated that the flavonoid pathway contributed substantially to the metabolomic separation between the two species.

### 2.5. Cluster Heatmap Analysis of Flavonoid- and Phenylpropanoid Pathway-Related Differential Metabolites

Flavonoids and phenylpropanoids are important bioactive components of *Apocynum*. In total, 39 differential metabolites related to the flavonoid and phenylpropanoid pathways were annotated, including 12 in roots, 13 in stems, and 14 in leaves. Hierarchical cluster analysis of these metabolites in each tissue was performed, and the results are shown in Figure 5a–c.

The heatmaps of roots, stems, and leaves showed differences in the relative abundance of flavonoid- and phenylpropanoid-pathway-related differential metabolites between the two species. Coniferin and Quercetin in stems were relatively higher in *A. venetum*, whereas Taxifolin and naringenin-O-dihexoside in leaves were relatively higher in *A. pictum*. The clear grouping of samples by species within each tissue heatmap reflected the reproducibility of the between-species differences at the level of individual flavonoid metabolites.

### 2.6. Between-Species Expression Abundance of Flavonoid- and Phenylpropanoid Pathway-Related Differential Metabolites

The between-species abundances of the flavonoid- and phenylpropanoid-pathway-related differential metabolites were compared statistically (Figure 6a–c).

Among the 12 flavonoid- and phenylpropanoid-pathway-related metabolites in roots, most showed significant overall differences between the two species. Sinapic acid and Epigallocatechin were significantly higher in *A. venetum*, whereas naringenin-O-dihexoside, Sinapoyl-(S)-malate, 3,4-Dihydroxy-5-methoxycinnamic acid and Pinocembrin were significantly higher in *A. pictum*; Hesperetin and Cyanidin 3-O-beta-D-sambubioside showed no significant overall differences. The opposite enrichment directions of individual metabolites highlight a coordinated but largely inverted flavonoid metabolism between the two species.

Among the 13 flavonoid- and phenylpropanoid-pathway-related metabolites in stems, Luteolin, Quercetin, Sinapic acid, Coniferin, Hesperetin, Sinapyl alcohol, and Methyleugenol were significantly higher in *A. venetum*, whereas 3,4,2′,4′,6′-Pentahydroxychalcone, L-Tyrosine, naringenin-O-dihexoside, 5-Hydroxyconiferyl alcohol, and 3,4-Dihydroxy-5-methoxycinnamic acid were significantly higher in *A. pictum*.

Among the 14 flavonoid- and phenylpropanoid-pathway-related metabolites in leaves, Cyanin, Caffeic acid, Butin, 5-Caffeoylshikimic acid, and 4-coumaroylshikimate were significantly higher in *A. venetum*, whereas 5-Hydroxyconiferyl alcohol, naringenin-O-dihexoside, and Taxifolin were significantly higher in *A. pictum*; Trans-Cinnamic acid, Leucocyanidin, and Catechin showed no significant overall differences.

### 2.7. Comparison of Flavonoid- and Phenylpropanoid Pathway-Related Differential Metabolites Across Stages and Tissues

For each tissue, the between-species abundances were compared by growth stage (Welch’s *t*-test), along with an overall comparison across the whole experimental period; the results are shown in Table 2, Table 3 and Table 4.

With respect to growth stage, the number and composition of metabolites with significant between-species differences varied with stage in each tissue. In roots, the number of significantly different metabolites was highest at the growth stage (6), followed by four at the mature stage and four at the seedling stage. Hesperetin became significantly higher in *A. pictum* at the mature stage (Δ = −1.123 ***). These stage-dependent changes indicate that the timing of sampling is a critical factor in comparisons of the two species, and that single-stage sampling may either overlook or overstate certain between-species differences.

Detected replicates (n = 3 per species, tissue and stage). Naringenin-O-dihexoside was detected in 2/3 *A. venetum* leaf replicates at the seedling stage and 3/3 at the growth and mature stages, and in 3/3, 3/3 and 3/3 *A. pictum* leaf replicates at the seedling, growth and mature stages, respectively; one of the three growth-stage replicates was nevertheless at the noise floor (0.01 in raw peak area), numerically indistinguishable from the imputed minimum. Leucocyanidin was detected in 3/3 *A. venetum* leaf replicates at all three stages and in 1/3, 3/3 and 2/3 *A. pictum* leaf replicates at the seedling, growth and mature stages, respectively. Missing values were filled with the minimum value of each feature; for these two compounds the leaf comparison should therefore be interpreted in terms of detection rather than as a difference between mean abundances.

In stems, the number of significantly different metabolites was highest at the seedling stage (10), followed by 8 at the growth stage and 4 at the mature stage. Luteolin, Quercetin, Hesperetin, Methyleugenol, and Coniferin were higher in *A. venetum*, with the most pronounced differences at the seedling stage; Coniferin was significantly higher in *A. venetum* at all three stages (Δ = 0.346 **, 0.314 *, and 0.210 **). The pronounced differences at the seedling stage suggest that young stem tissues are particularly responsive to the species-specific regulation of flavonoid metabolism.

In leaves, the number of significantly different metabolites was highest at the mature stage (10), followed by nine at the seedling stage, whereas only two were significant at the growth stage. 5-Caffeoylshikimic acid and Butin were higher in *A. venetum* at the seedling and mature stages, and 4-coumaroylshikimate was significantly higher at the mature stage (Δ = 1.367 **), whereas Taxifolin, Trans-Cinnamic acid, and naringenin-O-dihexoside were higher in *A. pictum*; for naringenin-O-dihexoside, *A. venetum* was close to the limit of detection in several leaf replicates (see the note to Table 4).

Naringenin-O-dihexoside was overall higher in *A. pictum* in roots, stems, and leaves (overall Δ = −0.786 *, −0.270 **, and −1.778 *, respectively; in leaves, naringenin-O-dihexoside was detected in only two of the three *A. venetum* seedling replicates and was close to the detection limit at the other stages, so the leaf comparison is reported as detected/not detected rather than as a difference between mean abundance). 5-Hydroxyconiferyl alcohol was higher in *A. pictum* in both stems (Δ = −0.300 ***) and leaves (Δ = −0.450 **). Coniferin (stems) was consistently higher in *A. venetum*.

## 3. Discussion

### 3.1. Medicinal Significance of Between-Species Flavonoid- and Phenylpropanoid-Related Differential Metabolites

Among the high-confidence (Level 2) annotations, *A. venetum* was relatively enriched in flavone/flavonol-type compounds (Luteolin and Quercetin). Among the tentative (Level 3) annotations, by contrast, *A. pictum* was relatively enriched in flavanone glycosides, such as naringenin-O-dihexoside, and phenylpropanoid compounds, such as 5-hydroxyconiferyl alcohol. This differentiation was observed in roots, stems, and leaves and is consistent with previous comparative analyses of the two species based on HPLC and metabolomics [14,15,18].

Quercetin and Luteolin in *A. venetum* possess antioxidant, anti-inflammatory, vasodilatory, and antihypertensive activities, consistent with the traditional effect of *Apocynum* leaves in cardiovascular protection [19,20]. These components were already enriched in *A. venetum* stems at the seedling stage, indicating that stems also provide a flavonoid basis relevant to cardiovascular protection. In contrast, *A. pictum* was relatively enriched in flavanone glycosides such as naringenin-O-dihexoside; its flavonoid composition is skewed toward flavanones and phenylpropanoids, complementing the flavone/flavonol-dominated metabolic profile of *A. venetum*. The early enrichment of Quercetin and Luteolin in *A. venetum* stems at the seedling stage suggests a developmentally coordinated accumulation of cardioprotective flavonoids, supporting the use of stems as an additional source of such active components.

Among the candidate metabolites screened, Coniferin was significantly higher in *A. venetum* stems at all three growth stages (Table 3). Naringenin-O-dihexoside was overall higher in *A. pictum* in roots, stems, and leaves, although the significance of the difference varied with growth stage and, in leaves, several *A. venetum* replicates fell below the limit of detection (Table 2 and Table 4; see the note to Table 4). These candidate markers were stable in direction of change across growth stages and thereby meet the reproducibility expected of chemical markers; they can serve as candidate markers for species identification and quality evaluation of *A. venetum* and *A. pictum*, pending confirmation by targeted quantitative analysis. The between-species differences in flavonoid biosynthesis may originate from genetic differences in the key enzyme genes of flavonoid synthesis and their expression regulation between the two species. Future studies combining transcriptome sequencing and molecular marker development could further resolve the molecular basis and translate the candidate markers into practically applicable methods for identification and quality control [21].

### 3.2. Distribution of Flavonoid- and Phenylpropanoid-Related Differential Metabolites Among Tissues and Evaluation of Medicinal Parts

Flavonoid metabolism showed marked tissue specificity, consistent with previous studies [15,22]. Roots showed a consistent enrichment of flavonoid biosynthesis but no significant enrichment of isoflavonoid biosynthesis at any growth stage, stems were dominated by flavone/flavonol and phenylpropanoid intermediates, and leaves covered the richest structural diversity of flavonoids (including anthocyanins, flavanones, flavanols, and flavanonols) and showed the most significant enrichment of the flavonoid biosynthesis pathway. This tissue-specific metabolic differentiation corresponds to the physiological functions of each tissue: roots emphasize the accumulation of flavonoid-type compounds, stems are closely associated with the phenylpropanoid metabolism linked to lignin synthesis, and leaves are the most active organ in flavonoid biosynthesis. Beyond the stem-associated monolignol-type metabolites, the phenylpropanoid pathway also contributed to the between-species differences in leaves through phenylpropanoid esters such as 5-caffeoylshikimic acid, 4-coumaroylshikimate, and Caffeic acid (Table 4), consistent with the significant enrichment of phenylpropanoid biosynthesis in leaves.

Both traditional use and the Chinese Pharmacopoeia designate *Apocynum* leaves for medicinal use [5,23]. In this study, leaves exhibited the most complete structural diversity of flavonoid compounds and the highest enrichment of the flavonoid biosynthesis pathway, consistent with their status as the traditional medicinal part. The diverse flavonoids detected in leaves, such as Apigenin 7-O-(6″-malonyl-apiosyl-glucoside) and Catechin, provide a material basis for the effects of *Apocynum* leaves in tranquilizing the mind, clearing heat, and promoting diuresis [3]. Moreover, the number of significantly different metabolites between species was highest in leaves at the mature stage. This consistency between the pharmacopoeial designation of leaves and the observed metabolomic pattern provides a chemical basis for the traditional medicinal use of *Apocynum* leaves.

Roots and stems also hold potential medicinal value. The potential activities of the root flavonoid-type components and the flavone/flavonol components in stems have been discussed in the interspecific comparison above. The clinical and pharmacological activities of *Apocynum* leaf extracts have been widely reported [8,18], and several chemical components with potential anti-diabetic activity have also been isolated and identified from the flowers [12]. The tissue-specific allocation of distinct flavonoid types among roots, stems, and leaves implies that the whole plant of both species can be rationally exploited, with each tissue serving as a dedicated source of particular bioactive flavonoids.

From the temporal perspective, key differential metabolites such as Coniferin in stems maintained stable between-species differences across all stages, indicating that the between-species differences at certain pathway nodes are temporally robust [16]. In summary, *A. venetum* and *A. pictum* can be differentially utilized according to the flavonoid composition of each tissue: leaves for the comprehensive utilization of multiple flavonoid types, stems as a supplementary source of flavonol/phenylpropanoid components, and roots for the targeted development of flavonoid-type active components [24,25]. The regulation of flavonoid biosynthesis in *Apocynum* is simultaneously influenced by tissue development and genetic background, and the specific regulatory nodes warrant further validation at the gene expression level [21]. Integrating such gene-level evidence would not only explain the observed metabolic differences but also facilitate the development of molecular markers for germplasm selection. With respect to growth stage, the number of metabolites with significant between-species differences changed markedly across growth stages: in leaves the largest number was observed at the mature stage (10), in roots at the growth stage (6), and in stems at the seedling stage (10) (Table 2, Table 3 and Table 4). This stage dependence suggests that the timing of harvest affects both the magnitude of the between-species differences and the resolving power of candidate markers: leaf material intended for medicinal use is best discriminated at the mature stage, whereas root-derived markers are most informative at the growth stage. These phenylpropanoid-related compounds were also stage-dependent: in stems, Coniferin was higher in *A. venetum* at all three growth stages, with the largest difference at the seedling stage (Δ = 0.346 **, Table 3), when the number of significantly different metabolites in stems was also highest (10); in leaves, 4-coumaroylshikimate (mature Δ = 1.367 **) and 5-caffeoylshikimic acid (seedling Δ = 1.373 ***, mature Δ = 0.337 *) were higher in *A. venetum*, indicating that phenylpropanoid esters contributed to the mature-stage discrimination of leaves.

## 4. Materials and Methods

### 4.1. Plant Materials and Cultivation

The cultivation of *A. venetum* and *A. pictum* followed the method described by Huang et al. [26]. On 15 December 2024, seeds were surface-sterilized with 0.1% potassium permanganate solution for 10 min, soaked in sterile water for 24 h, and sown in plug trays filled with vermiculite. The seedlings were irrigated with half-strength Hoagland nutrient solution and, 2 months later, were transplanted into a greenhouse for establishment. Seeds of both species were collected from the Tarim Basin, China, and identified by Prof. Jian-Min Cong.

### 4.2. Sample Collection

Roots, stems, and leaves of healthy *A. venetum* and *A. pictum* plants were collected at the seedling, growth, and mature stages on 15 March, 15 June, and 15 September 2025, respectively, with three biological replicates per stage. The samples were immediately frozen in liquid nitrogen and stored in an ultra-low-temperature freezer for subsequent metabolomics analysis.

### 4.3. Metabolite Extraction and LC-MS/MS Analysis

A 100 mg aliquot of each sample was placed in a 2 mL centrifuge tube together with a 6 mm grinding bead, and 400 µL of extraction solvent (methanol–water, 4:1, *v*/*v*) containing four internal standards (0.02 mg/mL L-2-chlorophenylalanine, etc.) were added. The mixture was homogenized in a frozen tissue grinder for 6 min (−10 °C, 50 Hz), extracted by low-temperature ultrasonication for 30 min (5 °C, 40 kHz), and allowed to stand at −20 °C for 30 min. After centrifugation at 13,000× *g* and 4 °C for 15 min, the supernatant was collected for analysis. A pooled quality control (QC) sample was prepared by mixing equal volumes of all sample extracts and was processed and analyzed in the same manner as the analytical samples.

Analysis was performed on an ultra-high-performance liquid chromatography system (Vanquish UHPLC; Thermo Fisher Scientific, Waltham, MA, USA) coupled to a high-resolution mass spectrometer (UHPLC-Q-Exactive HF-X; Thermo Fisher Scientific, Waltham, MA, ). Metabolites were separated on an ACQUITY UPLC BEH C18 column (100 mm × 2.1 mm i.d., 1.7 µm; Waters, Milford, MA, USA). Mobile phase A was 2% acetonitrile in water containing 0.1% formic acid, and mobile phase B was acetonitrile containing 0.1% formic acid; the flow rate was 0.40 mL/min, the column temperature was 40 °C, and the injection volume was 1 µL. The elution gradient was as follows: 0–3.5 min, 2–50% B; 3.5–6 min, 50–95% B; 6–6.5 min, 95% B; 6.5–6.6 min, 95–2% B; and 6.6–8 min, 2% B. The total run time was 8 min. The mass spectrometer was operated in positive and negative electrospray ionization (ESI) modes over an *m*/*z* range of 70–1050. The sheath and auxiliary gas flow rates were 50 and 13 arbitrary units, and the heater and capillary temperatures were 425 °C and 325 °C, respectively. The spray voltages were +3500 V (positive) and −3000 V (negative), and the normalized collision energies were 20, 40 and 60 eV. Full-MS and MS/MS resolutions were 60,000 and 7500, respectively, and data were acquired in data-dependent acquisition (DDA) mode. A QC sample was injected every 5–15 analytical samples to monitor the stability of the analysis.

### 4.4. Data Processing and Statistical Analysis

Raw data were processed with Progenesis QI v3.0 (Waters Corporation, Milford, MA, USA) for peak detection, extraction, alignment and integration. Metabolite annotation was performed by matching the MS and MS/MS spectra against the HMDB database (v5.0; http://www.hmdb.ca/ (accessed on 25 July 2026)), Metlin (v3.0; https://metlin.scripps.edu/ (accessed on 25 July 2026)) and the in-house plant-specific metabolite database (MJDBPM) of Majorbio Bio-Pharm Technology Co., Ltd. (Shanghai, China), with a mass error below 10 ppm and MS/MS spectral matching scores. Because annotation relied on spectral library matching rather than authentic standards, compounds are referred to as “annotated” throughout and were not confirmed with authentic reference compounds. Following the confidence-level framework of Schymanski (2014) [27] and Sumner (2007) [28], each annotation was assigned a confidence level. Features matched to reference MS/MS spectra in the spectral library were classified as Level 2 (putatively identified), whereas features assigned only by exact mass and in silico (theoretical) fragmentation were classified as Level 3 (tentative). No feature reached Level 1, because no authentic standard was used. Because annotation relied on spectral library matching, some assigned compounds that are biogenetically unexpected for the Apocynaceae (e.g., Fabaceae-type isoflavonoids/pterocarpans and other non-Apocynaceae compounds, including the Brassicaceae-typical ester sinapoyl-(S)-malate) were treated as tentative annotations [29,30]. Features with more than 20% missing values in each group were removed, and the remaining missing values were filled with the minimum value of each feature. Peak intensities were normalized using the sum normalization method, and variables with a QC relative standard deviation (RSD) > 30% were removed. The data were then log10-transformed. Principal component analysis (PCA, mean-centering) and partial least squares discriminant analysis (PLS-DA, Pareto scaling) were performed with the R package ropls (v1.6.2) using 7-fold cross-validation, and the models were validated by permutation testing (200 permutations) and group separation was assessed by ANOSIM. Metabolites with a variable importance in projection (VIP) value > 1 and *p* < 0.05 (Student’s *t*-test) were considered significantly differential, and their between-species abundance at each growth stage was further evaluated by Welch’s *t*-test. Pathway annotation and enrichment analysis were performed against the KEGG database using the Python package scipy.stats (v1.17.1) (Fisher’s exact test). The same screening criteria were applied across tissues and growth stages to ensure the comparability of the results. Because the number of features tested was large, a Benjamini–Hochberg (BH) FDR adjustment was also performed, and the complete *p* values and BH-FDR-adjusted values for all features are provided in Supplementary Table S2, so that every reported metabolite can be evaluated under either criterion.

## 5. Conclusions

This study used LC-MS/MS-based untargeted metabolomics to systematically compare the flavonoid- and phenylpropanoid-related differential metabolites in the roots, stems, and leaves of *A. venetum* and *A. pictum* at the seedling, growth, and mature stages. The results showed that the metabolite profiles of the two species differed significantly: *A. venetum* was relatively enriched in flavone/flavonol-type compounds, whereas *A. pictum* was relatively enriched in flavanone glycosides (such as naringenin-O-dihexoside) and phenylpropanoid compounds. Flavonoid metabolism exhibited marked tissue specificity: leaves showed the richest structural diversity of flavonoids and the highest enrichment of the flavonoid biosynthesis pathway, roots showed consistent enrichment of flavonoid biosynthesis but no significant enrichment of isoflavonoid biosynthesis, and stems were closely associated with the accumulation of flavonol/phenylpropanoid components. Among the 39 pathway-related differential metabolites, naringenin-O-dihexoside (in *A. pictum*) and Coniferin (in *A. venetum* stems) showed between-species differences that were stable in direction across growth stages, with significance varying by stage (Table 2, Table 3 and Table 4), and are proposed as candidate chemical markers for species identification, pending confirmation by targeted analysis. These findings elucidate the species-, tissue-, and stage-dependent accumulation patterns of flavonoids in the two *Apocynum* species at the metabolomics level and provide a scientific basis for the quality evaluation, species identification, and differentiated utilization of the whole-plant flavonoid active components.

## Figures and Tables

**Figure 1:**
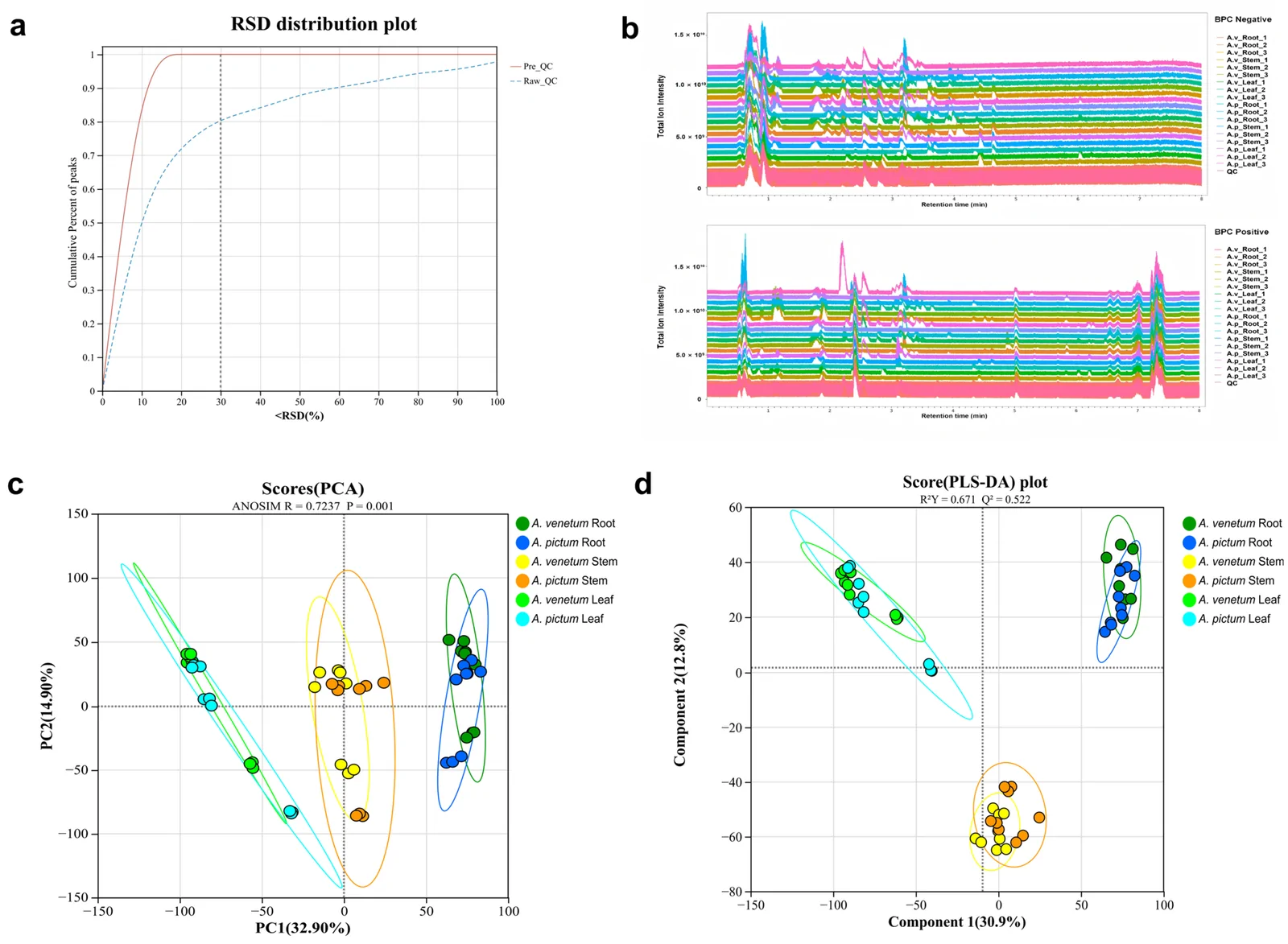
Quality control and multivariate statistical analyses of the metabolomics data of *A. venetum* and *A. pictum*. (**a**) Cumulative distribution of the RSD of the QC sample peaks before (Raw_QC) and after (Pre_QC) data preprocessing. (**b**) Base-peak chromatograms (BPC) in negative (upper) and positive (lower) ESI modes, with the sample and QC runs overlaid showing consistent peak shapes and retention times. (**c**) PCA score plot (PC1 = 32.90%, PC2 = 14.90%); ANOSIM R = 0.7237, *p* = 0.001. (**d**) PLS-DA score plot (R^2^Y = 0.671, Q^2^ = 0.522, 7-fold cross-validation; permutation Q^2^ intercept = −0.5111, *p* < 0.05), showing separation between *A. venetum* and *A. pictum* within each tissue.

**Figure 2:**
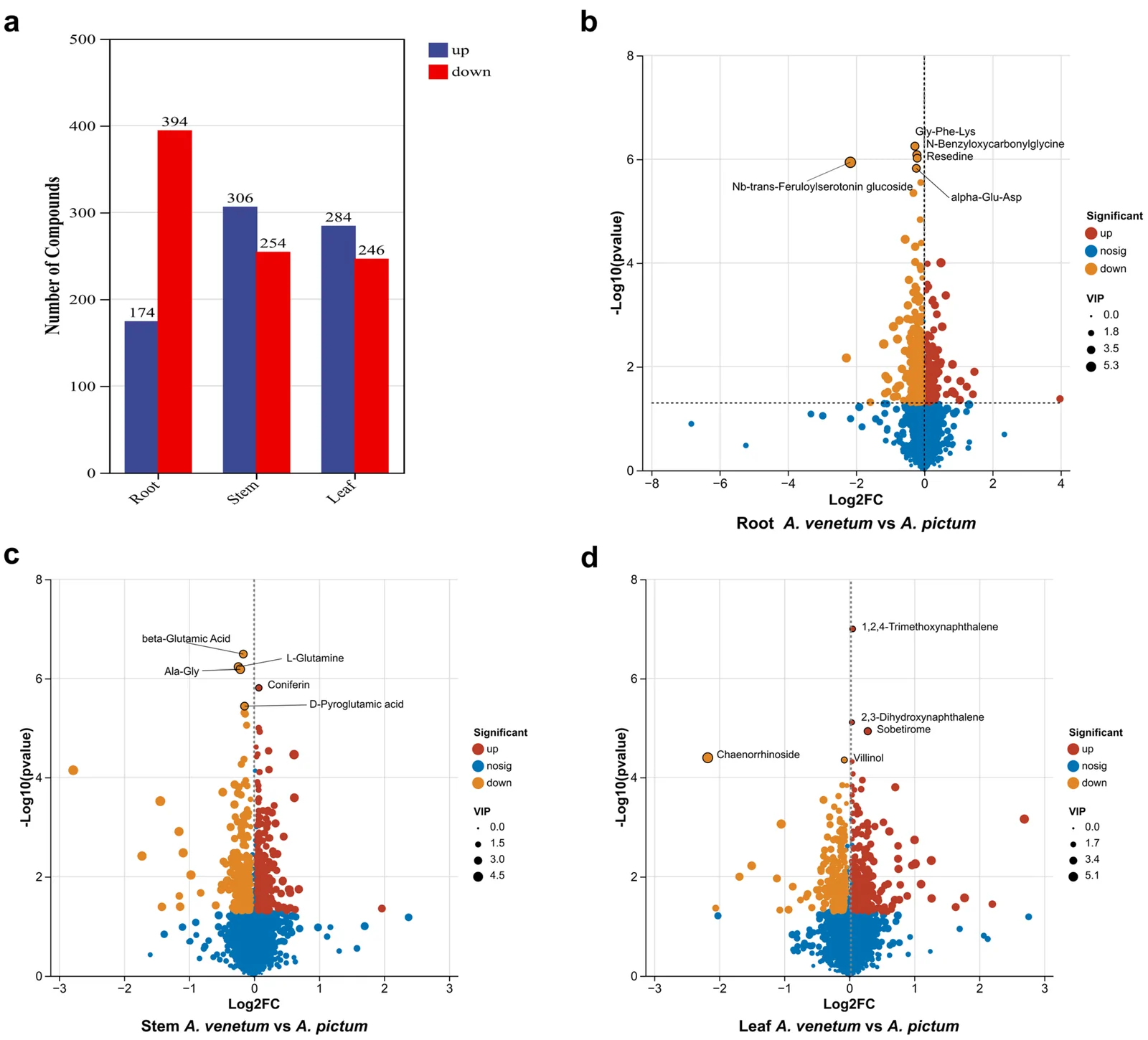
Comparison of differential metabolites between *A. venetum* and *A. pictum*. (**a**) Numbers of up- and down-regulated differential metabolites in root, stem and leaf; volcano plots of root (**b**), stem (**c**) and leaf (**d**). Positive log_2_FC values indicate higher abundance in *A. venetum*.

**Figure 3:**
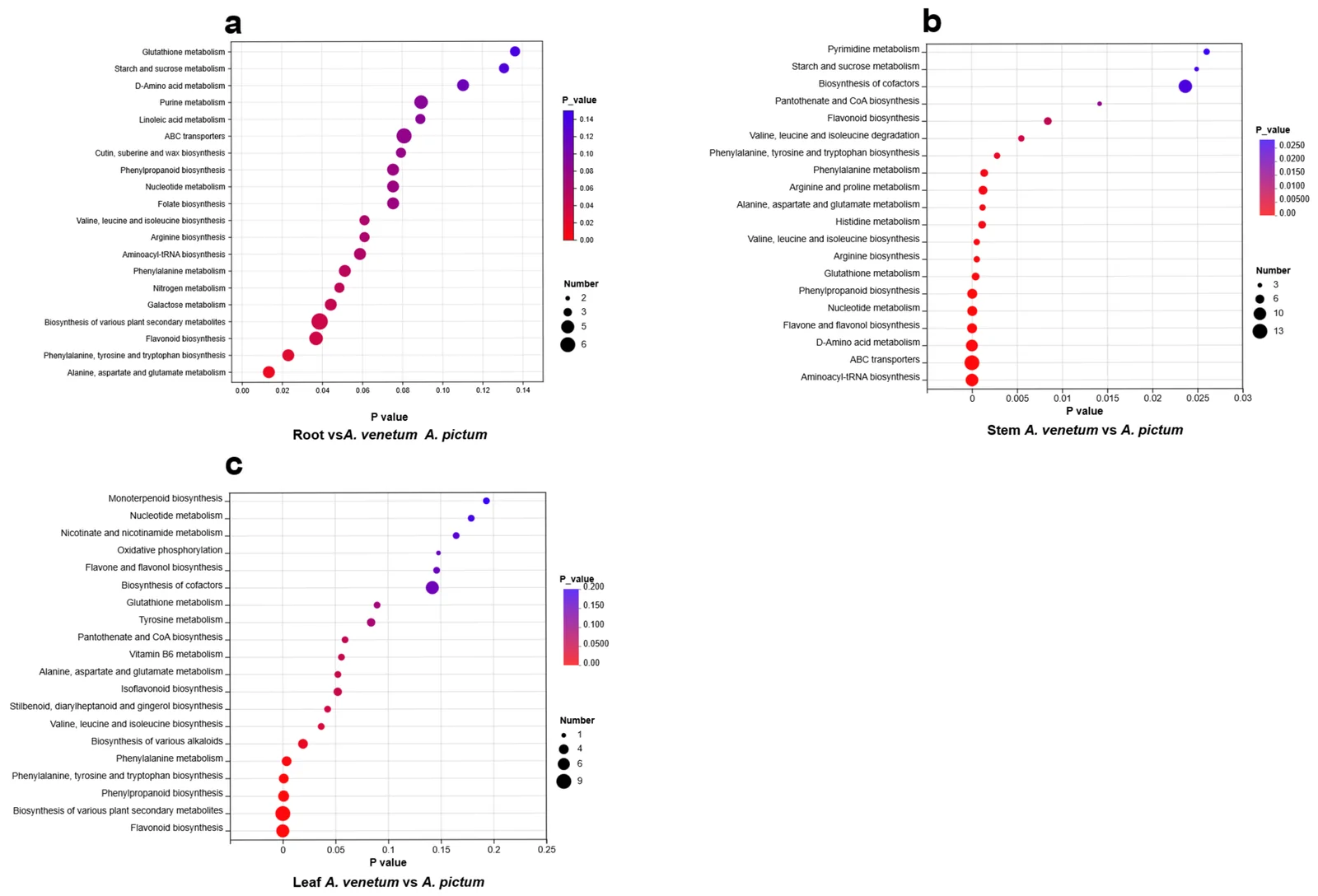
Bubble plots of KEGG pathway enrichment analysis of differential metabolites between *A. venetum* and *A. pictum* in the root (**a**), stem (**b**), and leaf (**c**).

**Figure 4:**
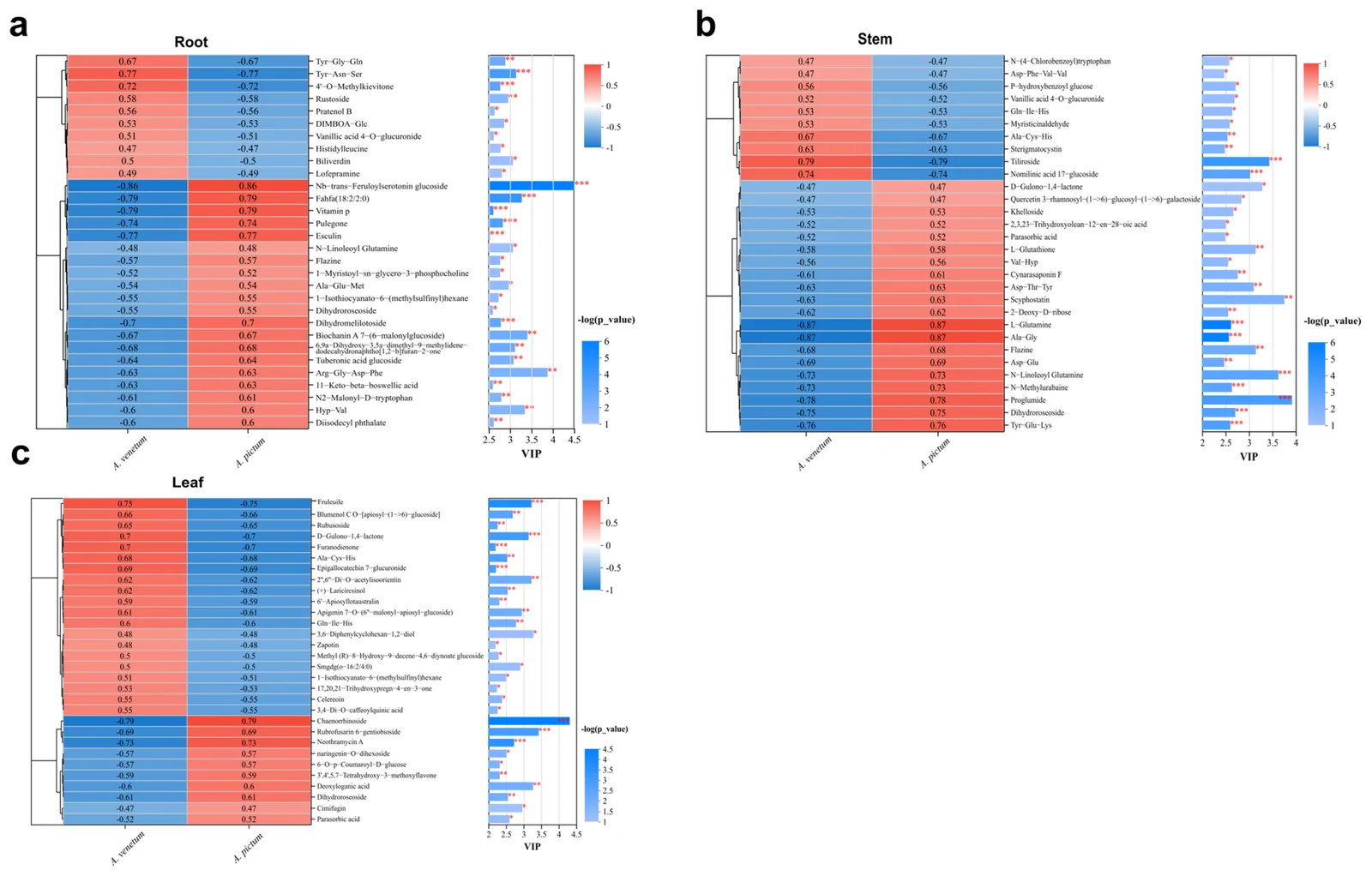
VIP analysis of differential metabolites between *A. venetum* and *A. pictum* in root (**a**), stem (**b**), and leaf (**c**); higher VIP values indicate greater contribution to the between-species separation.Asterisks indicate the significance of the between-species difference (Welch’s *t*-test: * *p* < 0.05, ** *p* < 0.01, *** *p* < 0.001).

**Figure 5:**
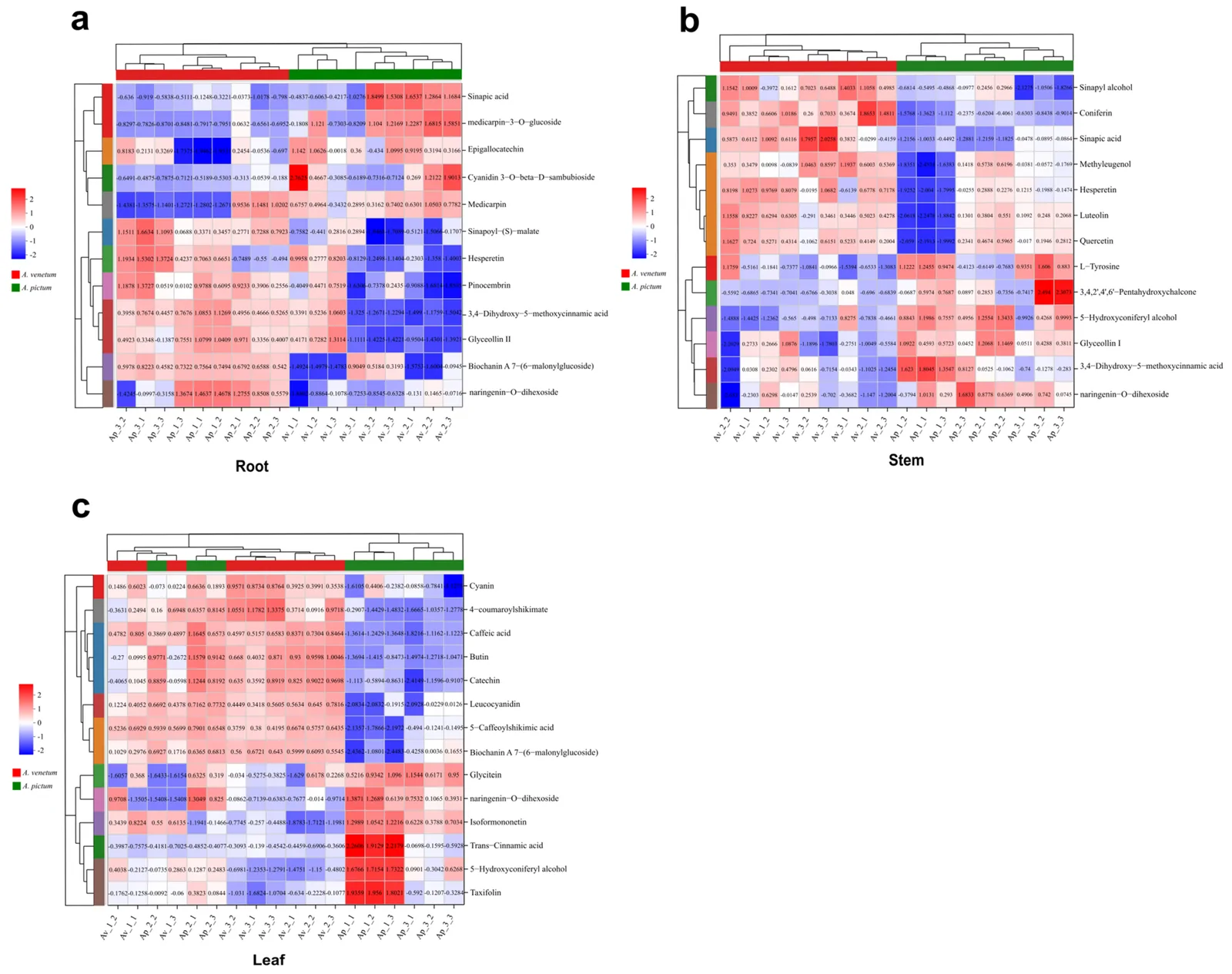
Hierarchical cluster heatmaps of differential metabolites involved in flavonoid and phenylpropanoid metabolism in the root ((**a**), 12 metabolites), stem ((**b**), 13 metabolites), and leaf ((**c**), 14 metabolites). Sample codes: Av and Ap denote *A. venetum* and *A. pictum*, respectively; the first digit indicates the growth stage (1, seedling; 2, growth; 3, mature) and the second digit the biological replicate.

**Figure 6:**
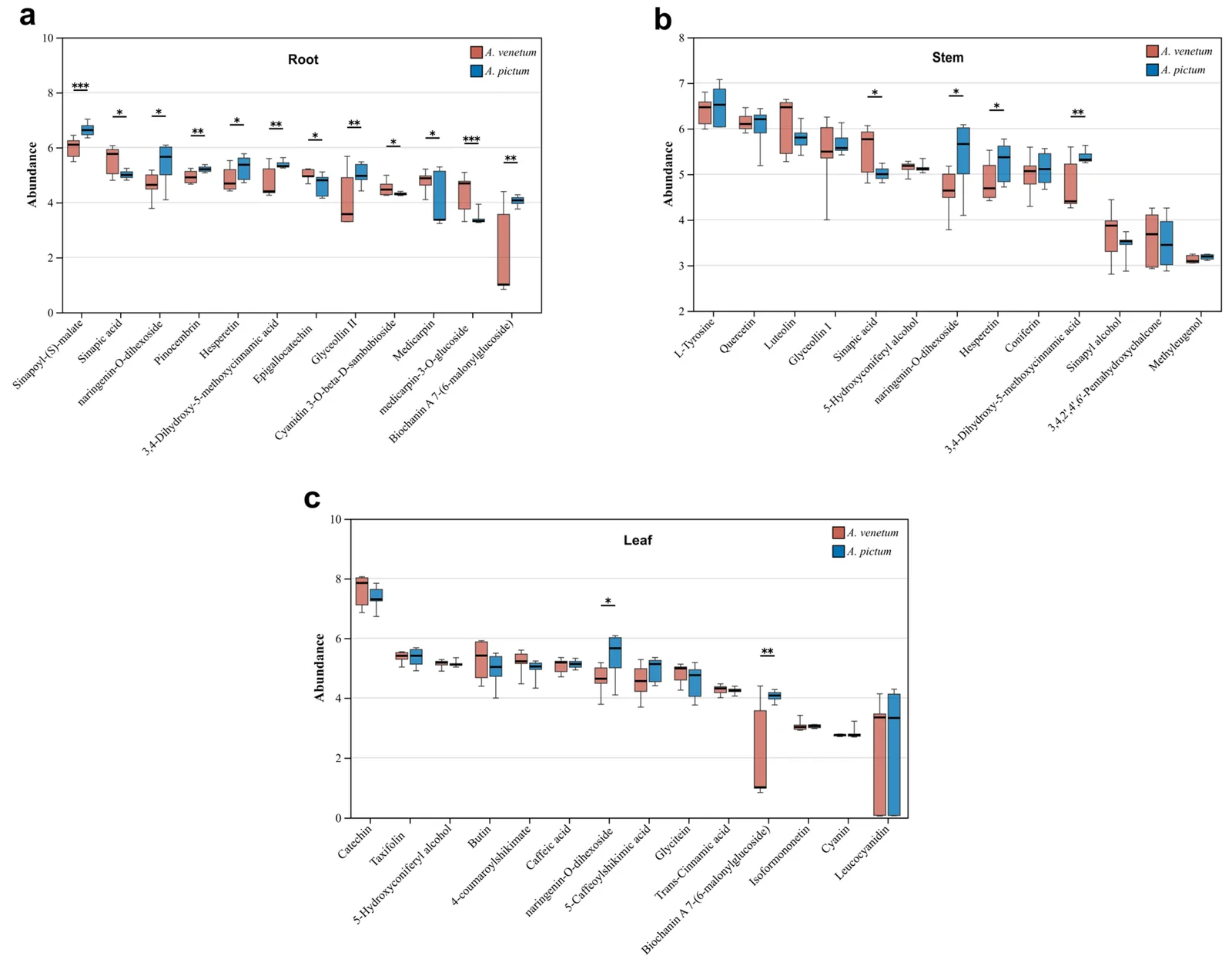
Boxplots of the relative abundance (log10-transformed normalized peak areas) of the flavonoid- and phenylpropanoid-pathway-related differential metabolites in the root ((**a**), 12 metabolites), stem ((**b**), 13 metabolites), and leaf ((**c**), 14 metabolites), comparing *A. venetum* and *A. pictum* within each tissue.Red boxes, A. venetum; blue boxes, A. pictum. Asterisks indicate the significance of the between-species difference (Welch’s *t*-test: * *p* < 0.05, ** *p* < 0.01, *** *p* < 0.001).

**Table 1:** Categories and numbers of annotated metabolites.

Category	Class	Number
Secondary metabolites	Terpenoids	511
	Flavonoids	479
	Phenolic acids and derivatives	255
	Steroids and derivatives	144
	Coumarins and derivatives	94
	Lignans and derivatives	72
	Indoles and derivatives	61
	Alkaloids and derivatives	41
	Quinones	40
	Tannins	21
	Stilbenes	18
	Vitamins	13
Primary metabolites	Lipids	472
	Amino acids and derivatives	366
	Carbohydrates and derivatives	281
	Organic acids and derivatives	146
	Nucleotides and derivatives	51
	Others	1973

**Table 2:** Comparison of flavonoid- and phenylpropanoid-pathway-related differential metabolites in roots between *A. venetum* and *A. pictum*.

Metabolite	Seedling			Growth			Mature			Overall
	*A. venetum*	*A. pictum*	Δ	*A. venetum*	*A. pictum*	Δ	*A. venetum*	*A. pictum*	Δ	Δ
medicarpin-3-O-glucoside (L3)	3.942 ± 0.679	3.312 ± 0.023	0.630	4.963 ± 0.170	3.585 ± 0.305	1.377 **	4.249 ± 0.818	3.301 ± 0.031	0.948	0.985 **
Sinapic acid (L2)	5.036 ± 0.041	5.117 ± 0.084	−0.081	5.855 ± 0.111	4.986 ± 0.225	0.869 *	5.599 ± 0.690	4.945 ± 0.079	0.655	0.481 *
Hesperetin (L2)	5.388 ± 0.173	5.342 ± 0.070	0.046	4.605 ± 0.306	4.789 ± 0.062	−0.184	4.572 ± 0.105	5.696 ± 0.078	−1.123 ***	−0.420
Epigallocatechin (L2)	5.081 ± 0.220	4.181 ± 0.042	0.900 *	5.007 ± 0.120	4.770 ± 0.166	0.237	4.946 ± 0.264	4.984 ± 0.111	−0.038	0.366 *
Cyanidin 3-O-beta-D-sambubioside (L2)	4.616 ± 0.333	4.291 ± 0.023	0.325	4.648 ± 0.171	4.374 ± 0.027	0.274	4.270 ± 0.013	4.279 ± 0.031	−0.010	0.196
Medicarpin (L3)	4.598 ± 0.433	3.365 ± 0.005	1.233 *	5.030 ± 0.170	5.206 ± 0.079	−0.176	4.735 ± 0.201	3.334 ± 0.123	1.401 **	0.820 *
Pinocembrin (L2)	5.134 ± 0.132	5.192 ± 0.107	−0.059	4.750 ± 0.111	5.190 ± 0.077	−0.440 **	4.920 ± 0.206	5.267 ± 0.157	−0.347	−0.282 **
Naringenin-O-dihexoside (L3)	4.418 ± 0.611	6.062 ± 0.039	−1.645 *	5.064 ± 0.101	5.692 ± 0.248	−0.628 *	4.569 ± 0.077	4.655 ± 0.489	−0.085	−0.786 *
Biochanin A 7-(6-malonylglucoside) (L3)	0.997 ± 0.014	4.171 ± 0.018	−3.174 ***	1.564 ± 1.224	4.002 ± 0.105	−2.438	3.937 ± 0.423	4.001 ± 0.261	−0.064	−1.892 **
Glyceollin II (L3)	5.251 ± 0.394	5.373 ± 0.154	−0.121	3.448 ± 0.232	5.034 ± 0.304	−1.586 **	3.395 ± 0.156	4.739 ± 0.285	−1.344 **	−1.017 *
3,4-Dihydroxy-5-methoxycinnamic acid (L2)	5.379 ± 0.195	5.563 ± 0.102	−0.183	4.320 ± 0.098	5.304 ± 0.016	−0.984 **	4.382 ± 0.025	5.325 ± 0.105	−0.943 **	−0.703 **
Sinapoyl-(S)-malate (L3)	6.190 ± 0.228	6.428 ± 0.067	−0.238	6.008 ± 0.297	6.578 ± 0.120	−0.569	5.840 ± 0.526	6.881 ± 0.132	−1.041	−0.616 ***

Values are log10-transformed normalized peak areas (relative abundance; mean ± SD); Δ = *A. venetum* − *A. pictum* on the log10 scale; Welch’s *t*-test; * *p* < 0.05, ** *p* < 0.01, *** *p* < 0.001.

**Table 3:** Comparison of flavonoid- and phenylpropanoid-pathway-related differential metabolites in stems between *A. venetum* and *A. pictum*.

Metabolite	Seedling			Growth			Mature			Overall
	*A. venetum*	*A. pictum*	Δ	*A. venetum*	*A. pictum*	Δ	*A. venetum*	*A. pictum*	Δ	Δ
3,4,2′,4′,6′-Pentahydroxychalcone (L2)	2.962 ± 0.018	3.834 ± 0.338	−0.872 *	3.009 ± 0.058	3.411 ± 0.414	−0.402	3.266 ± 0.277	4.538 ± 1.389	−1.272	−0.849 *
Luteolin (L2)	6.670 ± 0.044	5.581 ± 0.072	1.089 ***	6.671 ± 0.158	6.536 ± 0.084	0.135	6.449 ± 0.145	6.470 ± 0.028	−0.022	0.401 *
Sinapic acid (L2)	6.136 ± 0.044	5.824 ± 0.076	0.313 **	6.003 ± 0.097	5.759 ± 0.010	0.244 *	6.262 ± 0.170	5.980 ± 0.004	0.282	0.280 ***
Sinapyl alcohol (L2)	4.233 ± 0.104	4.111 ± 0.015	0.122	4.331 ± 0.054	4.217 ± 0.032	0.114 *	4.331 ± 0.062	3.949 ± 0.082	0.382 **	0.206 **
Hesperetin (L2)	6.626 ± 0.018	6.182 ± 0.016	0.443 ***	6.595 ± 0.011	6.505 ± 0.026	0.089 *	6.502 ± 0.133	6.468 ± 0.027	0.034	0.189 **
L-Tyrosine (L2)	6.347 ± 0.044	6.597 ± 0.024	−0.250 **	6.381 ± 0.203	6.328 ± 0.028	0.053	6.280 ± 0.116	6.602 ± 0.064	−0.323 *	−0.173 *
Quercetin (L2)	7.689 ± 0.048	6.843 ± 0.031	0.846 ***	7.699 ± 0.162	7.648 ± 0.059	0.051	7.620 ± 0.126	7.558 ± 0.049	0.061	0.320 *
Methyleugenol (L2)	4.595 ± 0.092	3.759 ± 0.172	0.836 **	4.760 ± 0.052	4.739 ± 0.107	0.021	4.977 ± 0.068	4.522 ± 0.030	0.455 **	0.437 *
Coniferin (L3)	5.886 ± 0.054	5.541 ± 0.039	0.346 **	6.013 ± 0.078	5.698 ± 0.033	0.314 *	5.845 ± 0.039	5.635 ± 0.024	0.210 **	0.290 ***
Glyceollin I (L3)	5.869 ± 0.112	5.908 ± 0.080	−0.039	5.442 ± 0.202	5.930 ± 0.155	−0.488 *	5.483 ± 0.180	5.808 ± 0.049	−0.325	−0.284 **
Naringenin-O-dihexoside (L3)	5.593 ± 0.100	5.633 ± 0.156	−0.040	5.191 ± 0.191	5.803 ± 0.123	−0.612 *	5.503 ± 0.109	5.662 ± 0.076	−0.159	−0.270 **
5-Hydroxyconiferyl alcohol (L3)	4.746 ± 0.097	5.175 ± 0.048	−0.429 **	4.781 ± 0.111	5.193 ± 0.099	−0.412 **	4.947 ± 0.177	5.005 ± 0.217	−0.058	−0.300 ***
3,4-Dihydroxy-5-methoxycinnamic acid (L2)	4.498 ± 0.063	4.877 ± 0.064	−0.379 **	4.012 ± 0.151	4.500 ± 0.138	−0.488 *	4.365 ± 0.119	4.321 ± 0.090	0.043	−0.274 *

Values are log10-transformed normalized peak areas (relative abundance; mean ± SD); Δ = *A. venetum* − *A. pictum* on the log10 scale; Welch’s *t*-test; * *p* < 0.05, ** *p* < 0.01, *** *p* < 0.001.

**Table 4:** Comparison of flavonoid- and phenylpropanoid-pathway-related differential metabolites in leaves between *A. venetum* and *A. pictum*.

Metabolite	Seedling			Growth			Mature			Overall
	*A. venetum*	*A. pictum*	Δ	*A. venetum*	*A. pictum*	Δ	*A. venetum*	*A. pictum*	Δ	Δ
Cyanin (L2)	5.083 ± 0.214	4.573 ± 0.733	0.510	5.170 ± 0.017	5.085 ± 0.262	0.085	5.536 ± 0.033	3.968 ± 1.117	1.567	0.721 *
Trans-Cinnamic acid (L2)	4.151 ± 0.051	4.882 ± 0.050	−0.730 ***	4.183 ± 0.045	4.200 ± 0.011	−0.016	4.236 ± 0.042	4.243 ± 0.074	−0.007	−0.251
Caffeic acid (L2)	5.774 ± 0.036	5.400 ± 0.014	0.374 **	5.816 ± 0.013	5.803 ± 0.077	0.013	5.765 ± 0.020	5.394 ± 0.079	0.371 *	0.253 **
Butin (L2)	4.617 ± 0.162	3.808 ± 0.240	0.809 *	5.461 ± 0.029	5.501 ± 0.096	−0.039	5.220 ± 0.178	3.761 ± 0.171	1.459 ***	0.743 *
5-Caffeoylshikimic acid (L2)	6.219 ± 0.046	4.847 ± 0.115	1.373 ***	6.237 ± 0.025	6.263 ± 0.052	−0.026	6.113 ± 0.013	5.776 ± 0.108	0.337 *	0.561 *
Glycitein (L3)	1.575 ± 1.374	3.743 ± 0.356	−2.168	2.405 ± 1.444	2.442 ± 1.484	−0.037	2.341 ± 0.305	3.811 ± 0.326	−1.470 **	−1.225 *
Leucocyanidin (L3)	4.623 ± 0.328	1.271 ± 2.063	3.352	5.268 ± 0.208	5.374 ± 0.098	−0.106	4.863 ± 0.207	2.691 ± 2.277	2.173	1.806
Isoformononetin (L3)	4.750 ± 0.059	4.896 ± 0.031	−0.147 *	4.213 ± 0.087	4.540 ± 0.215	−0.327	4.483 ± 0.064	4.744 ± 0.041	−0.260 **	−0.244 *
5-Hydroxyconiferyl alcohol (L3)	4.883 ± 0.114	5.420 ± 0.010	−0.537 *	4.469 ± 0.176	4.863 ± 0.056	−0.394 *	4.456 ± 0.112	4.875 ± 0.162	−0.419 *	−0.450 **
Biochanin A 7-(6-malonylglucoside) (L3)	4.750 ± 0.141	1.634 ± 1.125	3.116 *	5.319 ± 0.042	5.436 ± 0.042	−0.118 *	5.372 ± 0.083	4.355 ± 0.437	1.016	1.338
Catechin (L2)	6.806 ± 0.159	6.358 ± 0.160	0.448 *	7.429 ± 0.044	7.456 ± 0.098	−0.027	7.264 ± 0.163	5.968 ± 0.492	1.296 *	0.572
Naringenin-O-dihexoside (L3)	1.415 ± 2.189	4.123 ± 0.652	−2.708	1.502 ± 0.789	2.724 ± 2.385	−1.222	1.666 ± 0.536	3.070 ± 0.507	−1.404 *	−1.778 *
Taxifolin (L2)	4.890 ± 0.028	5.860 ± 0.040	−0.970 ***	4.794 ± 0.133	5.021 ± 0.098	−0.228	4.342 ± 0.175	4.781 ± 0.113	−0.439 *	−0.546 *
4-coumaroylshikimate (L3)	4.234 ± 0.288	3.546 ± 0.368	0.687	4.388 ± 0.244	4.420 ± 0.184	−0.032	4.775 ± 0.077	3.408 ± 0.173	1.367 **	0.674 **

Values are log10-transformed normalized peak areas (relative abundance; mean ± SD); Δ = *A. venetum* − *A. pictum* on the log10 scale; Welch’s *t*-test; * *p* < 0.05, ** *p* < 0.01, *** *p* < 0.001.

## Data Availability

The raw MS data are available in the MetaboLights repository (https://www.ebi.ac.uk/metabolights/) (accessed on 20 September 2026) under the identifier MTBLS15526.

## Supplementary Materials

The following supporting information can be downloaded at https://www.mdpi.com/article/10.3390/molecules31193401/s1: Supplementary Table S1: Annotation overview for all detected metabolites (metabolite name, molecular formula, adduct, measured *m*/*z*, retention time, Metlin and HMDB match scores, mass error (ppm), and confidence level); Supplementary Table S2: statistics for the differential metabolites in roots, stems, and leaves (*p* values, Benjamini–Hochberg FDR-adjusted values, OPLS-DA and PLS-DA VIP values, fold change/log_2_FC) together with the corresponding annotation evidence (KEGG, HMDB, and Metlin/HMDB match scores); Supplementary Figure S1: MS/MS mirror plots for Coniferin and naringenin against reference spectra, together with a statement on the annotation of naringenin-O-dihexoside.

## Abbreviations

The following abbreviations are used in this manuscript:

Abbreviation	Full Name
PCA	Principal component analysis
PLS-DA	Partial least squares discriminant analysis
OPLS-DA	Orthogonal partial least squares discriminant analysis
VIP	Variable importance in projection
UHPLC	Ultra-high-performance liquid chromatography
LC-MS/MS	Liquid chromatography–tandem mass spectrometry
QC	Quality control
RSD	Relative standard deviation
BPC	Base peak chromatogram
KEGG	Kyoto Encyclopedia of Genes and Genomes
FC	Fold change
PC	Principal component
