# Supplementary material for: Comparative Untargeted Metabolomics of Flavonoid- and Phenylpropanoid-Related Differential Metabolites in Different Tissues and Growth Stages of Apocynum venetum and Apocynum pictum
Source: Molecules. 2026 Sep 24;31(19):3401. doi: 10.3390/molecules31193401 (PMC13665917; doi:10.3390/molecules31193401)
Supplement: Supplementary file 1 [file molecules-31-03401-s001.zip › Supplementary Figure S1.pdf]

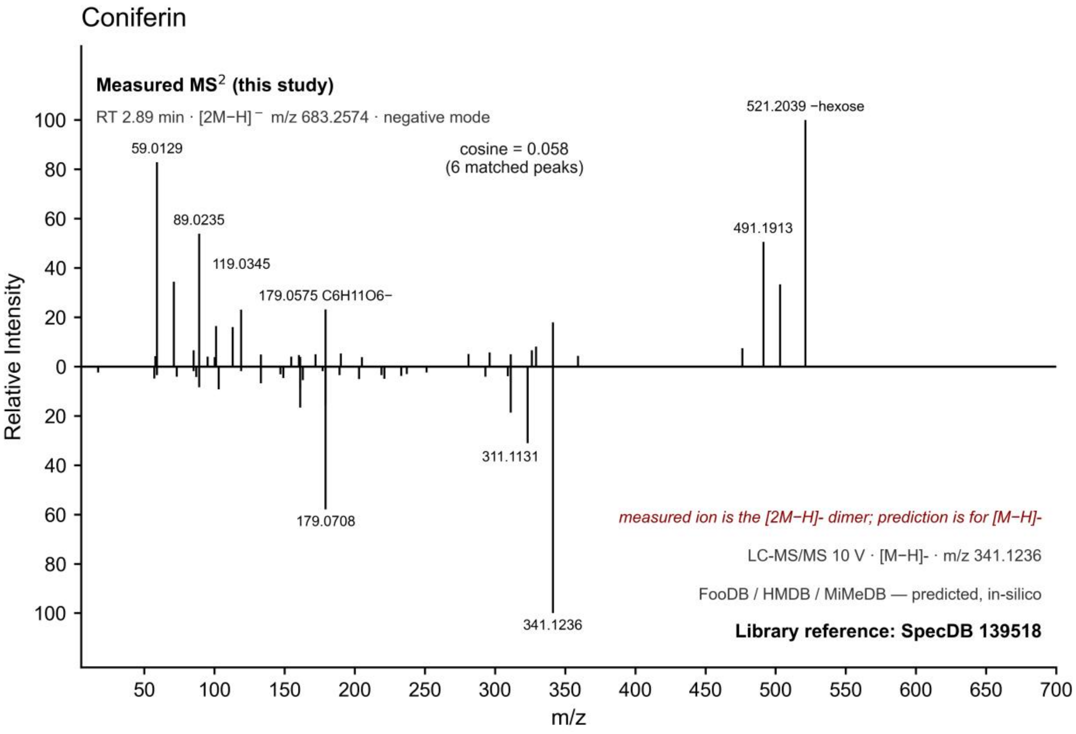

(a) Coniferin

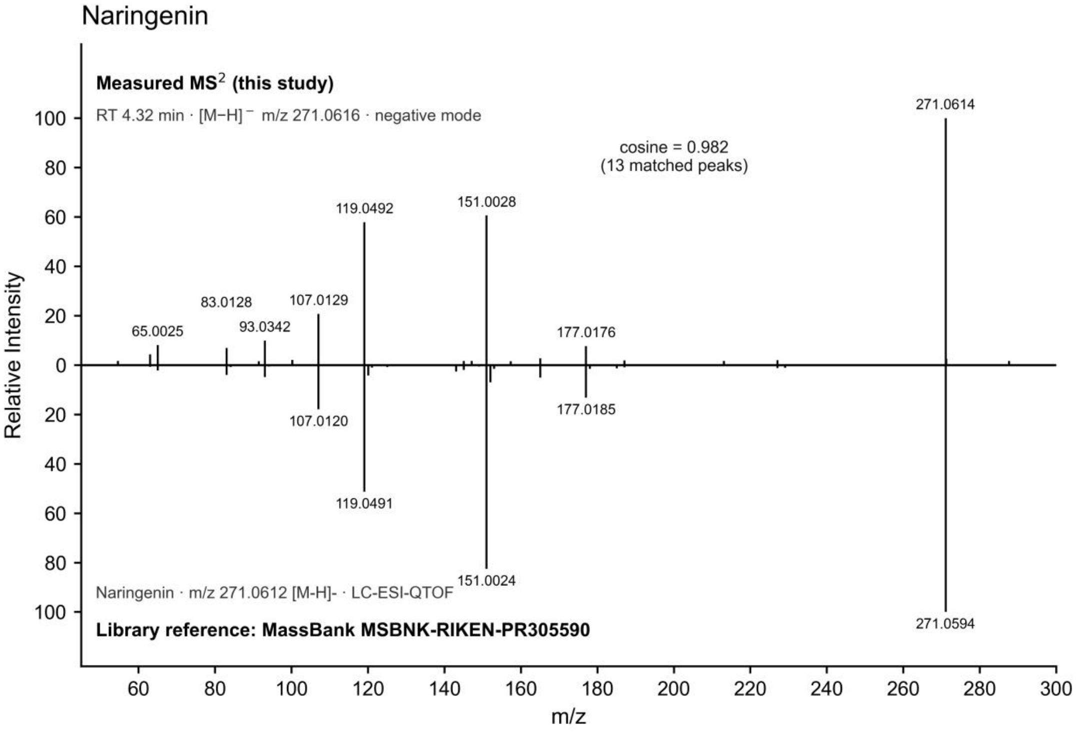

(b) Naringenin

Supplementary Figure mirror plots.

(a) Coniferin: MS/MS of the feature at m/z 683.2574 ([2M−H]⁻, RT 2.89 min) in A. venetum stems (upper) versus the predicted LC-MS/MS spectrum of coniferin (HMDB0013682 / FDB015497 / MMDBc0052970), negative mode, 10 V ([M−H]⁻, m/z 341.1236; lower). The two halves derive from different precursor ions (dimer versus monomer) and are therefore compared at the fragment level only: the hexose-derived fragments at m/z 179.0575 and 161.0466 agree with the prediction to within 1.9 and 1.6 mDa, whereas the coniferyl-alcohol aglycone ion predicted at m/z 179.0708 was not observed.

(b) Naringenin (the aglycone of naringenin-O-dihexoside): MS/MS of the feature at m/z 271.0616 ([M−H]⁻, RT 4.32 min; upper) versus MassBank MSBNK-RIKEN-PR305590 ([M−H]⁻, m/z 271.0612; lower); cosine similarity 0.982 (13 matched peaks).

Note on naringenin-O-dihexoside. No MS/MS mirror plot can be provided for this compound. Across all 61 negative-mode samples, none of the MS/MS acquisitions falling within its [M−H]⁻ (m/z 579.17) or [M+FA−H]⁻ (m/z 625.18) windows contained a naringenin-type fragment ion (m/z 271.0612 or 459.1134), and the platform reports a fragmentation-library score of 0 for this feature. **The hexose identities and the interglycosidic linkage of naringenin-O-dihexoside therefore cannot be assigned from the data**; its annotation rests on accurate mass, retention time and the library name match, and is reported at Level 3 (tentative).
